# Diagnostic work-up and advancement in the diagnosis of gastroenteropancreatic neuroendocrine neoplasms

**DOI:** 10.3389/fsurg.2023.1064145

**Published:** 2023-03-06

**Authors:** Apostolos Koffas, Alexandros Giakoustidis, Apostolis Papaefthymiou, Petros Bangeas, Dimitrios Giakoustidis, Vasileios N Papadopoulos, Christos Toumpanakis

**Affiliations:** ^1^ Centre for Immunobiology, Blizard Institute, Barts and the London School of Medicine and Dentistry, Queen Mary University of London, London, United Kingdom; ^2^ 1st Department of Surgery, General Hospital Papageorgiou, School of Medicine, Faculty of Medical Sciences, Aristotle University Thessaloniki, Thessaloniki, Greece; ^3^ Pancreaticobiliary Medicine Unit, University College London Hospitals (UCLH), London, United Kingdom; ^4^ Centre for Gastroenterology, Neuroendocrine Tumour Unit, ENETS Centre of Excellence, Royal Free Hospital, London, United Kingdom

**Keywords:** neuroendocrine tumor, ki67 proliferation index, chromogranin A, gut peptide hormones, NETest, somatostatin receptor scintigraphy, 68-gallium-DOTA-Octreotate

## Abstract

Neuroendocrine neoplasms (NENs) are a heterogeneous group of neoplasms ranging from well-differentiated, slowly growing tumors to poorly differentiated carcinomas. These tumors are generally characterized by indolent course and quite often absence of specific symptoms, thus eluding diagnosis until at an advanced stage. This underscores the importance of establishing a prompt and accurate diagnosis. The gold-standard remains histopathology. This should contain neuroendocrine-specific markers, such as chromogranin A; and also, an estimate of the proliferation by Ki-67 (or MIB-1), which is pivotal for treatment selection and prognostication. Initial work-up involves assessment of serum Chromogranin A and in selected patients gut peptide hormones. More recently, the measurement of multiple NEN-related transcripts, or the detection of circulating tumor cells enhanced our current diagnostic armamentarium and appears to supersede historical serum markers, such as Chromogranin A. Standard imaging procedures include cross-sectional imaging, either computed tomography or magnetic resonance, and are combined with somatostatin receptor scintigraphy. In particular, the advent of ^111^In-DTPA-octreotide and more recently PET/CT and ^68^Ga-DOTA-Octreotate scans revolutionized the diagnostic landscape of NENs. Likewise, FDG PET represents an invaluable asset in the management of high-grade neuroendocrine carcinomas. Lastly, endoscopy, either conventional, or more advanced modalities such as endoscopic ultrasound, capsule endoscopy and enteroscopy, are essential for the diagnosis and staging of gastroenteropancreatic neuroendocrine neoplasms and are routinely integrated in clinical practice. The complexity and variability of NENs necessitate the deep understanding of the current diagnostic strategies, which in turn assists in offering optimal patient-tailored treatment. The current review article presents the diagnostic work-up of GEP-NENs and all the recent advances in the field.

## 1. Introduction

Neuroendocrine neoplasms (NEN) are rare and heterogeneous tumors that are phenotypically similar and derive from the diffuse neuroendocrine cell system. These neoplasms demonstrate a rising prevalence and incidence. This is likely a result of the deeper and better understanding of these tumors, but also of the advent and integration of more advanced diagnostic means (1–4). In general, NENs exhibit slow growth and often absence of specific symptoms, which may in turn delay the diagnosis until at an advanced stage, when overt symptoms may develop. Among the several distinct sites of origin, gastroenteropancreatic NENs (GEP NENs) represent the commonest subtype, accounting for nearly 60% of all NENs. Among these, small bowel- (SBNEN) and pancreatic- NENs (pNEN) are the most frequent (5–8).

In addition to the variable primary sites, tumor heterogeneity is also evident by their variable biologic behavior. Often these tumors run a “benign” course with no ostensible disease progression and excellent prognosis. However, non-uncommonly, they may also be truly malignant, associated with an aggressive course, poor prognosis and a very limited life expectancy, mimicking other cancers (9).

This complexity and variability of NENs necessitate the deeper and better understanding of the current diagnostic armamentarium and strategic approach, and integration in clinical practice of all novel diagnostic tools. This in turn is pivotal to determine the optimal (tailored) treatment, including accurate selection of surgical candidates. The diagnostic cascade should be initiated once there is clinical suspicion. Initial work-up involves assessment of serum Chromogranin A and, in selected patients, measurement of gut peptide hormones. Recently, the measurement of multiple NEN-related transcripts or the detection of circulating tumor cells has been introduced and will play a key role, and seems to be superior to historical serum markers, such as Chromogranin A. Cross-sectional imaging, combined with somatostatin receptor scintigraphy and PET scan will complement the diagnostic approach and assist in disease stratification. Ultimately, the gold-standard of the diagnosis remains histopathology. The present review discusses the diagnostic work-up of GEP-NENs and presents all the novel diagnostic means that emerged over the last years. Table 1 summarizes current diagnostic modalities and their clinical utility.

**Table 1:** Current diagnostic tools in neuroendocrine neoplasms (NENs).

Modality	Indication	Strengths	Limitations
**Histopathology (tissue diagnosis)**	– To be pursued in all NENs, when feasible	– Gold standard for diagnosis	– Expert pathologist input recommended
**Biomarkers**			
Serum Chromogranin A (CgA)	– At diagnosis and during follow-up	– Well-studied biomarker – Can be used in functional and non-functional NENs	– Moderately sensitive, variable specificity – Not useful prognosticator – False positive results due to several factors – international standard for CgA assay lacks
Urinary 5-hydroxyindoleacetic acid (5-HIAA)	– At diagnosis and during follow-up – Particularly useful in patients with carcinoid syndrome	– Well-studied biomarker – Significant sensitivity and specificity especially in carcinoid syndrome	– Not useful prognosticator – Dietary restrictions prior to urine collection – Falsely elevated or low due to various factors
Gut Peptide Hormones (insulin, gastrin, VIP, glucagon, somatostatin)	– Used for functional NENs, especially pancreatic and duodenal	– Inappropriate elevation of the appropriate, specific serum hormonal marker required for diagnosis	– Should be interpreted with caution, and within a relevant clinical context – Various factors affect levels
**Cross-sectional imaging**			
Contrast-enhanced computed tomography (CT)	– Backbone of diagnosis, staging, follow-up and assessment of treatment response	– Broadly available – Well established modality – Best modality to assess vascular infiltration – Useful in the pre-operative setting	– Radiation exposure – Variable sensitivity – Less accurate in the diagnosis of gastric, duodenal, rectal and colonic NENs (still important for staging)
Contrast-enhanced magnetic resonance imaging (MRI)	– Similar to CT – Modality of choice, or complementary to CT	– Not contraindicated in patients allergic to iodine contrast – No radiation exposure – Superior to CT in assessing bone, brain, or abdominal disease – Superior to CT when hepatocyte-specific contrast is used	– Less available than CT – Contraindicated in patients with metallic implants
**Nuclear Medicine and Hybrid Imaging**			
^68^Gallium-DOTA-peptides	– Investigation of choice for well-differentiated NENs	– Mean sensitivity and specificity: 88%–93% and 88%–95%, respectively – Superior to cross-sectional imaging in bone metastases – Sensitive in detecting even subtle lymph node or small peritoneal metastases – Unaffected by the use of somatostatin analogs before examination – Lower exposure to radiation than classical scintigraphy	– Still not broadly available – More expensive than other modalities
^18^FDG PET/CT	– More useful in high-grade poorly differentiated neuroendocrine carcinomas (NEC)	– More sensitive than other modalities in detecting even subtle high-grade NECs	– Still not broadly available – More expensive than other modalities – Falsely positive results in active inflammation or infection
**Endoscopy**			
Gastroscopy/Colonoscopy	– Investigation of choice for gastric, duodenal, rectal and colonic NENs	– Allows biopsy of neoplasm – Primary NEN may be resected when indicated	– Invasive procedure – Associated with adverse events, in particular in frail patients – Biopsies may be misleading as NENs are subepithelial lesions
Endoscopic Ultrasound (EUS)	– Indicated in gastric, duodenal, and pancreatic NENs – Useful for diagnosis and staging – FNB should be preferred over FNA	– Increased sensitivity and specificity – Enables detection of previously unidentified tumors – Permits tissue diagnosis and histological evaluation (superior to conventional endoscopy)	– Invasive procedure – Associated with adverse events, in particular in frail patients – Depends on endoscopists skills
Small Bowel Capsule Endoscopy (SBCE)	– May have a role in detecting multifocal SBNENs pre-operatively or metastatic disease of unknown primary	– Enables detection of primary NEN or multiple NENs in small bowel (variable sensitivity) – -Could determine extent of resection	– Risk of capsule retention – Relevant expertise required – Further research required
Balloon Enteroscopy (BE)	– May have a role in detecting multifocal SBNENs pre-operatively or metastatic disease of unknown primary – Complementary to SBCE	– Enables detection of primary NEN or multiple NENs in small bowel (variable sensitivity) – Could determine extent of resection	– Invasive procedure – Associated with adverse events, in particular in frail patients – Relevant expertise required – Further research required

### 1.1. Clinical presentation

Not uncommonly, GEP-NENs are incidentally discovered. The rate of such presentation varies; for instance, in pNENs not producing hormones incidental diagnosis can exceed 50% (10), whereas it can be as high as 80% in the case of appendiceal NENs (11). Often NENs cause non-specific symptoms, such as abdominal pain or discomfort, weight loss, change in bowel habits or diarrhea. These symptoms are often attributed to other causes, such as gastritis, irritable bowel syndromes, or other relevant disorders, before a diagnosis is established. In contrast to such presentation, however, NENs may also overproduce hormones, such as 5-hydroxytryptamine (5-HT, serotonin), that results is associated symptoms. Such tumors are termed functional NENs, in contrast to non-functional NENs. For instance, the “carcinoid syndrome” is a syndrome that is mostly present in SBNENs, in the presence of hepatic or retroperitoneal metastases. This is caused due to 5-HT reaching the systemic circulation. As a result, patients present with a variety of symptoms, often precipated by a variety of foods, alcohol, stress, ot other triggers. Most commnonly subjects present with paroxysmal flushing, chronic diarrhea, wheezing and less frequently carcinoid heart disease (CHD), among others. Analogous to this, the secretion of other hormones by this subgroup of NENs, termed functionally active, such as insulin, gastrin, vasoactive intestinal peptide, glucagon, somatostatin, and others, can lead to specific syndromes, which are discussed later in more detail (6, 8). Table 2 summarizes functional pNENs and their respective presentation.

**Table 2:** Functional pancreatic neuroendocrine tumor syndromes.

	Biologically active peptide secreted	Tumor location	Associated with MEN-1, %	Main symptoms/signs
**Insulinoma**	Insulin	Pancreas (>99%)	4–5	Hypoglycaemic symptoms
**Zollinger-Ellison syndrome**	Gastrin	Duodenum (70%); Pancreas (25%); Other sites (5%)	20–25	Abdominal pain; peptic ulcer disease; diarrhoea; oesophageal symptoms (reflux)
**VIPoma (Verner-Morrison syndrome)**	Vasoactive intestinal peptide	Pancreas (90%, adult)	6	Profuse watery diarrhoea; hypokalaemia; dehydration
**Glucagonoma**	Glucagon	Pancreas (100%)	1–20	Dermatitis (necrolytic migratory erythema); glucose intolerance; weight loss; deep vein thrombosis
**Somatostatinoma**	Somatostatin	Pancreas (55%); duodenum/jejunum (44%)	45	diabetes mellitus; cholelithiasis; diarrhoea (steatorrhea)

## 2. Pathology

Histopathological confirmation represents the gold standard for the diagnosis of NENs and is recommended by the European Neuroendocrine Tumor Society (ENETS) (12). Tissue diagnosis should be pursued when clinically feasible. It should be noted that a biopsy is deemed superior to a fine needle aspirate (FNA) when, this is feasible (12, 13). This is of particular interest in the context of Endoscopic Ultrasound (EUS), where if not enough material is available, there is a risk of under-grading the tumor (6, 14, 15). In a recent study by our study group, data of patients who underwent EUS-guided tissue sampling of suspicious pancreatic lesions over a 13-year period were analyzed. Lesions underwent EUS-FNA or FNB sampling, or a combination of the two, and the accuracy and safety of different EUS-guided sampling methods for confirmed pNENs were investigated. Diagnostic yield of EUS-FNA and EUS-FNB alone, including the inadequate specimens, was 77.5 % (95 % CI: 68.9 %– 86.2 %) and 85.4 % (95 % CI: 74.6% – 96.2 %), respectively, whereas the combination of both sampling modalities established the diagnosis in over 95% of cases. Diagnostic sensitivity among the adequate samples for EUS-FNA, EUS-FNB and for the combination of the two methods was 88.4 % (95 % CI: 80.9% – 96.0 %), 94.3 % (95 % CI: 86.6% – 100 %) and 100 % (95 % CI: 100 %– 100%). These findings clearly illustrated that EUS-FNB improves diagnostic sensitivity and provides further information than cytological assessment alone, in patients with pNENs (16).

When a NEN is considered or clinically suspected, in addition to the conventional histopathological analysis, immunohistochemistry should be performed, to assess the tumor phenotype and Ki-67. Immunohistochemical staining with synaptophysin, and Chromogranin A (CgA) is also required. Ki-67 is a cell proliferation–associated nuclear marker, that is critical in assessing the differentiation of NENs, and as a result their respective course. CgA is a protein commonly secreted by neuroendocrine tumor cells (17).

The AJCC (American Joint Committee on Cancer), ENETS, UICC (International Union for Cancer Control), WHO (World Health Organization) developed a series of systems to classify NENs. Similar to other neoplasms, these classification systems are used to stage the disease, and are essential for treatment selection and prognostication (18–25). In particular, the WHO proposes a universal definition system for neuroendocrine neoplasia based on differentiation and proliferative grading. Table 3 summarises the novel WHO NEN classification.It integrates the mitotic count, and most importantly the nuclear antigen Ki-67, as markers of the proliferation activity of these neoplasms. Ki67 is more accurate and reproducible than the mitotic index (24, 25). Historically, a Ki-67 > 20% was believed to define poorly differentiated neoplasms, indicating an overall unfavorable prognosis. However, emerging evidence indicated that there is a distinct category of well-differentiated grade 3 neuroendocrine tumors (NETs), that is clearly different from the very aggressive poorly differentiated grade 3 neuroendocrine carcinomas (NECs), the latter associated with an unfavorable prognosis (19–21). More recently, the 5th edition of the WHO Classification of Endocrine Tumors was published, termed Classification of Endocrine and Neuroendocrine Tumors. This up-to-date classification system integrates this emerging evidence. In particular, the novel WHO 2022 system describes NECs, epithelial poorly differentiated neoplasms, composed of cells with severe cellular atypia and severely deranged molecular/genetic profiles, that broadly retain neuroendocrine markers. These NECs are further subclassified in small or large cell types, and Ki-67 is >20%, often >70%. This is in contrast to well differentiated grade 3 NETs. This is shown to have clinical implications on prognosis: Grade 3 NECs for instance were shown to have a 4 months' shorter median survival than G3 NETs and responded better to platinum-based chemotherapy (26, 27).

**Table 3:** WHO 2022 classification system for neuroendocrine tumors.

Neuroendocrine Neoplasm	Classification	Diagnostic Criteria
Well-differentiated neuroendocrine tumor (NET)	Grade 1	< 2 mitoses/2 mm^2^ and/or Ki67 < 3%
	Grade 2	2–20 mitoses/2 mm^2^ and/or Ki67 3%–20%
	Grade 3	> 20 mitoses/2 mm^2^ and/or Ki67 > 20%
Poorly differentiated neuroendocrine carcinoma (NEC)	Small cell NEC	> 20 mitoses/2 mm^2^ and/or Ki67 > 20% (often > 70%), and small cell cytomorphology
	Large cell NEC	> 20 mitoses/2 mm^2^ and/or Ki67 > 20% (often > 70%), and large cell cytomorphology

## 3. Biomarkers used in the diagnosis of neuroendocrine tumors

### 3.1. Classical blood and urine biomarkers

#### 3.1.1. Chromogranin A

Over the years, a considerable number of biomarkers have been integrated in clinical practice, and are used for diagnostic purposes, but also to follow-up patients with established disease. The most important among them is the general serum biomarker CgA. This is an acid glycoprotein present in the secretory dense core granules of most neuroendocrine cells. It is also secreted from neuroendocrine-derived tumors, including GEP-NENs, pheochromocytomas, and others. Of note, both functional and non-functional NENs may result in elevated CgA levels (28). CgA is a moderately sensitive marker, whereas specificity largely relies upon the type and tumor burden (for instance, specificity of approximately 100% has been reported in metastatic tumors). In particular, specificity of assays ranges from 68 to 100% and sensitivity ranges from 42%–93%, depending upon tumor primary site, grade, or disease burden (29). Significantly elevated CgA levels are unlikely to be encountered in other disease than NENs, with the exception maybe of patients receiving protein pump inhibitors (PPIs) (30–34). Although this is a marker that has been well validated for diagnostic and follow-up purposes, CgA cannot steadily be used for prognostication (35, 36).

In addition, CgA should be interpreted with caution as several conditions may lead to falsely positive elevation. Reasons for false positive CgA elevation include renal disease, Parkinson disease, uncontrolled hypertension, pregnancy, hypergastrinemia, chronic atrophic gastritis, among others. In addition, treatment with antisecretory medications, especially PPIs have been associated with falsely elevated CgA levels, and PPIs should be interrupted, leaving a clearance of at least 3 half-lives, prior to testing, where this is possible and safe for the patient. Analogous to this, steroid treatment or glucocorticoid excess can lead to upregulation of CgA. A limitation of CgA is also the fact that a recognized international standard for CgA assay is not available and variations in assay types may influence results. It is thus recommended that reference laboratories should be preferred when available, and that serial measurements should be performed using the same assay (28).

#### 3.1.2. Urinary 5-hydroxyindoleacetic acid

As already discussed, NENs may secrete 5-hydroxytryptamine (5-HT, serotonin) and other hormones, and in some subjects particularly with SBNENs this may mediate the carcinoid syndrome. Urinary 5-hydroxyindoleacetic acid (5-HIAA) is the urinary metabolite, following breakdown of 5-HT. Urinary 5-HIAA has proven of great value, and has been integrated in the diagnosis and follow-up of patients with carcinoid syndrome (28). The sensitivity and specificity of this marker has been reported to be 70% and 90%, respectively. Midgut NENs, such as SBNENs, produce more serotonin than the rest of the NENs, and it is when 5-HIAA is most useful. It should also be noted that urinary 5-HIAA levels depend upon the respective volume of the neoplasm and thus could be normal in individuals with no metastases (37, 38). Like CgA, there is no sound scientific evidence supporting the role of urinary 5-HIAA for prognosis. To increase accuracy, specific dietary restrictions should be followed prior to urine collection. Falsely low urinary 5-HIAA levels may be encountered in cases of impaired kidney function or on haemodialysis. Lastly, one needs to consider that urinary 5-HIAA levels may be falsely elevated in cases of malabsorptive disease, not treated. Examples include untreated coeliac disease, tropical sprue, Whipple disease, etc (28).

A less common manifestation of carcinoid syndrome is CHD, which affects nearly 20% of patients with “carcinoid syndrome”. In CHD, plaque-like, fibrous endocardial thickening of the cardiac valves develops. Patients with CHD have a poor prognosis. This is due to the gradual development of heart valve dysfunction and finally progressive heart failure. CHD is believed to be caused by this same tumor secretion of vasoactive hormonal products. Bhattacharyya et al. prospectively followed-up more than two hundred fifty patients with carcinoid syndrome for a median follow-up of 29 months. 44 of the included individuals either developed *de novo* CHD or exhibited deterioration in the pre-existing valvular dysfunction. This was associated with a synchronous elevation in the median levels of urinary 5-HIAA 5-HIAA levels of more than 300 μmol/24 h were reported to independently predict CHD development or progression, among other factors (39).

#### 3.1.3. N-terminal pro-brain natriuretic peptide

N-terminal pro-brain natriuretic peptide (NT-proBNP) is a natriuretic peptide, and it is an established diagnostic test for heart failure and its management. In the context of NENs, it should be used to screen for CHD, with proven and well-established value (40). Bhattacharyya et al. reported that in patients with carcinoid syndrome and valvular involvement, NT–proBNP was significantly higher than in patients without CHD. A cutoff level of 260 pg/ml was reported to be 92% sensitive and 91% specific to diagnose CHD in subjects with carcinoid syndrome (41). It is recommended that this biochemical marker should be routinely included in the diagnostic assessment and subsequent follow-up of patients with NENs and carcinoid syndrome.

#### 3.1.4. Gut peptide hormones

In functional tumors, measurement of specific hormones is appropriate [glucagon in glucagonoma, vasoactive intestinal peptide (VIP) in VIPoma, gastrin in gastrinoma, insulin in insulinoma, etc.]. There are two distinct clinical syndromes that need to be exceptionally presented: gastrinoma and Zollinger Ellison Syndrome (ZES); and insulinoma.

Gastrinomas are usually located in the duodenum or pancreas, secrete gastrin, and cause a clinical syndrome known as ZES. This results from hyperproduction of gastric acid by the parietal cells of the stomach, triggered by gastrin hypersecretion from the NEN. This in turn causes peptic ulcer disease, abdominal pain and chronic diarrhoea and malabsorption. The most common presentation of ZES is with duodenal ulcers, peptic ulcer symptoms, GERD symptoms or ulcer complications and diarrhea. Conversely, multiple ulcers or ulcers in unusual locations are a less frequent presenting feature than in the past. ZES should be suspected in cases of recurrent, severe or familial peptic ulcer disease, in particular when H. pylori is not detected or other risk factors are absent. In addition, peptic ulcer disease that is resistant to treatment, or associated with severe GORD or severe complications, should also prompt the physician to consider ZES (42). It should be noted that hypergastrinemia can occur much more frequently outside the context of a ZES cause, such as hypo- or achlorhydria secondary to chronic atrophic gastritis, pernicious anaemia, helicobacter pylori, or due to the use of proton-pump inhibitors (8). For ZES to be confidently diagnosed, inappropriately elevated fasting serum gastrin (FSG) level should be shown in the presence of gastric acid secretion. ZES can be diagnosed when FSG is more that 10-fold elevated and the gastric pH <2. However, in about 60% these requirements are not present (43), and additional tests are required, i.e., secretin test (44). It is acknowledged that the diagnosis of ZES is becoming more challenging, primarily due to increasing unreliability of commercial gastrin assays; the lack of availability of secretin to perform secretin provocative tests, where indicated; and the widespread use of PPIs (8).

Insulinoma is a rare pancreatic NEN (pNEN) that secretes insulin. This secretion is not properly regulated by glucose, and as a result Insulinomas continuously and inappropriately secrete insulin causing hypoglycaemia. Typically, patients with such tumors develop hypoglycaemia while fasting or during exercise, which improve by eating (8). In a consensus report from the US Endocrine Society, the proposed criteria for the diagnosis of Insulinoma were as follows: endogenous hyperinsulinism documented by the finding of symptoms, signs, or both; with plasma concentrations of glucose <55 mg/dl (3.0 mmol/litre), insulin ≥ 3.0 *μ*U/ml (18 pmol/litre), C-peptide ≥0.6 ng/ml (0.2 nmol/litre), and proinsulin ≥5.0 pmol/litre (45).

Most pNENs occur sporadically in a non-inherited fashion. Nevertheless, a variable proportion of them can emerge as part of an inherited syndrome. Multiple Endocrine Neoplasia type 1 (MEN1) is the most important inherited syndrome that accounts for up to 30% of gastrinomas and <5% of Insulinomas. This is an autosomal-dominant genetic condition involving the development of multiple tumors, arising from neuroendocrine cells. These tumors more frequently occur in endocrine glands, mainly the parathyroids, GEP system and pituitary gland (8). The diagnosis of MEN1 in patients with a functional pNEN is frequently markedly delayed (5–9.5 years) (46, 47). Patient diagnosed with ZES should be routinely screened for MEN1, due to the association between the two conditions. Relevant guidelines recommend that parathormone level in the serum, ionized calcium levels and prolactin are routinely performed at diagnosis, and then annually in these patients. Additionally, if a patient is diagnosed with insulinoma before the age of 20 or with multiple insulinomas at any age, MEN1 should also be suspected, and patient screened (8, 42). Overall, we would recommend that all patients with functional pNENs are screened, as above, despite some paucity of relevant data.

## 4. The role of contrast-enhanced imaging and nuclear imaging in the diagnosis of neuroendocrine neoplasms

Computed Tomography (CT) with contrast of the neck-chest-abdomen and pelvis, including three-phase CT of the liver, represents the cornerstone for the diagnosis, staging and follow-up of NENs. Magnetic resonance imaging (MRI) with contrast should be the examination of choice to assess the liver, pancreas, brain and bone, when possible. It is recommended that somatostatin receptor imaging is used for staging, follow-up, and on a pre-operative basis; ^68^Ga-DOTATATE is recommended when available. Lastly, ^18^FDG-PET/CT is of greater value in cases of higher glucose metabolism and less somatostatin receptor expression. Therefore, we recommend that ^18^FDG-PET/CT is considered for high grade NENs, mainly G3 tumors (48–52).

### 4.1. Cross-sectional imaging

#### 4.1.1. Contrast-enhanced computed tomography

CT scan represents the backbone of the NEN diagnosis; and is also broadly used for staging, surveillance and monitoring treatment response. Its' use is well-established as a result of its diagnostic accuracy and broad and steady availability. CT is the best modality to assess vascular infiltration and is very helpful in the pre-operative setting. The sensitivity and specificity for CT to diagnose individuals with NEN ranges from 61 to 93% and 71 to 100%, respectively (48).

In particular, the diagnostic accuracy of CT for the diagnosis of pNEN ranges from 69%–94% (53–56). For the diagnosis of small bowel NENs, CT enteroclysis exhibits sensitivity of up to 85% and 97%, respectively (57–59). CT enterography is similar to CT enteroclysis, primarily differing in the method of contrast administration. The sensitivity of both methods is comparable (60). Gastric (gNENs), duodenal (dNENs), rectal (rNENs) and NENs of the colon are often diagnosed by endoscopy, either conventional or EUS. Therefore, CT has a limited role for the diagnosis of these tumors, but should be used for staging (48). CT sensitivity and specificity for diagnosing liver metastases ranges from 75%–100% and 83%–100%, respectively (61–64). Careful consideration is required, however, as CT scan cannot steadily differentiate NEN liver metastases from liver metastases originating from other malignancies (48). Additionally, CT is less accurate in detecting smaller lesions (< 1cm) and bone metastases (sensitivity <60%) and this limitation needs to be considered (65–67).

#### 4.1.2. Contrast-enhanced magnetic resonance imaging

MRI is increasingly available and has the advantage of no exposure to radiation. Additionally, as is the case of other types of cancer, MRI appears superior to CT in assessing bone, brain, or abdominal disease, and may be preferred as the imaging modality of choice, or complementary to CT. In particular, MRI has higher tissue resolution than CT and should be preferred for assessing bone metastases. In addition, on patients who are allergic to iodine contrast agents, MRI should also be the preferred modality (68). Furthermore, diffusion weighted imaging (DW imaging) allows detection of subtle neoplastic tissue changes and is highly sensitive in detecting NEN-related liver metastases (48).

In the assessment of pNENs, tumor volume affects the accuracy of MRI. In particular, it is 70% sensitive for primary NENs larger than 2.5 cm, and this sensitivity decreases for lesions < 1.5 cm (69, 70). The accuracy of DW-MRI for the detection of primary NENs and metastatic disease is comparable to PET/CT (71–73). MRI is helpful in surgical planning and assessment of the relationship of the tumor to the main pancreatic duct if enucleation is planned (74). Several features of the neoplasms, such as its size and shape; enhancement pattern, vascular invasion, and involvement of lymph nodes can assist in determining tumor grade (74). MRI also enables additional sequences for pancreatic neoplasms; and in particular MRCP should be used to determine the anatomical relationship between the tumor and the pancreatic and common bile ducts (48).

The use of hepatocyte-specific contrast media renders MRI scan superior to CT in characterizing liver lesions (48, 74). In a prospective study that compared MRI, CT, and somatostatin receptor scintigraphy (SRS), MRI was reported to detect more metastasic sites compared to the other modalities. In particular, the respective sensitivity for detecting liver metastases was 95.2%, 78%, and 49.3% (75). If hepatic surgery is considered, MRI of the liver should be considered for better assessment of the hepatic tumor load prior to the surgical intervention.

Lastly, in the assessment of SBNENs, a recent study by Dohan et al., reported that the overall sensitivity of MR-enterography for small bowel NENs detection was 74% (95% CI: 54%–89%) on a per-lesion basis and 95% (95% CI: 74%–100%) on a per-patient basis, providing direct evidence of the diagnostic value of MRI in this setting too (76).

#### 4.1.3. The role of imaging in assessing carcinoid heart disease

Echocardiography (ECHO) is of paramount importance in the diagnosis of CHD and is the gold standard in determining severity of the condition. It is a prerequisite that only physicians experienced in its use are involved. Diffuse thickening of the valve leaflets; isolated thickening of a single valve leaflet without significant reduction in leaflet mobility; or the development of valvular regurgitation, may all be seen in CHD. 3-dimensional trans-thoracic-ECHO or trans-oesophageal ECHO may be preferred to examine the pulmonary and tricuspid valves (41). ENETS recommend echocardiography to be performed at baseline and then six monthly to annually in relevant patients (77).

Cardiac magnetic resonance imaging (CMRI) and cardiac computed tomography (CCT) are useful additional modalities, and can complement ECHO. The former is an excellent tool when echocardiographic windows are poor or when structures such as the pulmonary valve cannot be visualised. It also allows measurement of heart metastases and provides information on invasion of extra-cardiac structures (41). CCT can assist in examining the heart valves and right ventricular size and function; the coronary arteries before heart surgery; and depict myocardial metastases and their relationship with the affected valve(s) (78, 79).

### 4.2. Nuclear medicine and hybrid imaging

Somatostatin is a cyclic peptide that exerts strong regulatory effects in the body. The action of this protein is mediated through membrane-bound receptors. These receptors are expressed in high volumes in neuroendocrine cells, and currently five subclasses 1–5 have been cloned (sst1–sst5). These somatostatin receptors are also expressed in high volumes in NENs (80, 81). The landscape was revolutionised following the advent of PET/CT; and novel tracers have been developed, including somatostatin analogs (SSAs), such as ^68^Ga-DOTA, and metabolic markers, such as ^18^F-FDG (82, 83). DOTATOC OTANOC, and DOTATATE are the main DOTA-peptides that bind to somatostatin receptors. As a result, they are currently broadly used both in the diagnostic/staging cascade, but also for therapeutic purposes, i.e. Peptide receptor radionuclide therapy (PRRT) (84, 85). PET/CT with ^68^Ga-DOTA-peptides is more sensitivity than cross-sectional imaging with CT or classical scintigraphy for detecting well-differentiated tumors (52, 86). Conversely, ^18^F-FDG PET/CT is used for less differentiated NETs, due to the presence of increased glucose in these tumors. This also illustrates the increased propensity for more aggressive course and poor prognosis (87).

#### 4.2.1. The advent of ^68^Ga-DOTA-peptides

Historically, ^111^In-pentetreotide (Octreoscan™) represented the mainstay of SRS. ^68^Ga-DOTA-SSAs has superseded and replaced classical scintigraphy in an increasing number of healthcare settings, owing to the greater accuracy and lower exposure to radiation, and it is now considered the investigation of choice for well-differentiated NENs. Nevertheless, primarily due to financial limitations, Octreoscan™ still represents the backbone of scintigraphy in many centers, especially in healthcare settings with restricted resources. At present, we recommend that SRS should only be used only when PET/CT imaging is unavailable. Overall, the sensitivity of ^111^In-pentetreotide scintigraphy for the detection of these neoplasms ranges from 60%–80% and the specificity from 92%–100% (88–92). Conversely, ^68^Ga-DOTA-SSAs exhibits greater diagnostic accuracy, albeit small variations reported in the literature. In recent systematic reviews and meta-analyses comprising overlapping studies, mean sensitivities and specificities for NEN detection varied from 88%–93% and 88%–95%, respectively. ^68^Ga-DOTA-SSAs is superior to cross-sectional imaging in the detection of bone metastases, that are often subtle. Likewise, lymph node metastases may be characterised, and the detection of small peritoneal metastases is facilitated by ^68^Ga-DOTA-SSAs (93–96).

Yang et al. in their metanalysis included ten studies comprising 416 patients with NENs. The pooled sensitivity and sensitivity of ^68^Ga-DOTATOC in the diagnosis of NENs was 93% (95% confidence interval [CI] 89%–96%) and 85% (95% CI 74%–93%), respectively. The pooled sensitivity and specificity of ^68^Ga-DOTATATE PET in diagnosing NENs was 96% (95% CI 91%–99%) and 100% (95% CI 82%–100%), respectively (93). In a recent retrospective study of patients with pNENs across three tertiary UK NET referral centers ^68^Ga-DOTA PET/CT was assessed. It was reported that the findings of ^68^Ga-DOTA PET/CT imaging provided extra information in more than 50% of the studied subjects and had an impact on management decisions in nearly 40% (97). These studies clearly illustrate that ^68^Ga-DOTA PET/CT significantly upgraded and enhanced our diagnostic armamentarium. The quality of this diagnostic modality was also found to be unaffected by the use of SSAs before the examination, a key advantage. Thus, it is recommended against discontinuing short-acting SSAs before the examination (98).

#### 4.2.2. The role of PET/CT in high-grade disease

In contrast to ^68^Ga-DOTA PET/CT imaging, ^18^FDG PET/CT is generally better in the context of high-grade disease, indicating likely a more aggressive course. With increasing tumor proliferation, somatostatin receptor expression declines and so does uptake on SRS or ^68^Gallium-DOTA-SSAs (99, 100). Conversely, these lesions generally become more avid on ^18^FDG PET/CT with increasing proliferation. Therefore, ^18^FDG PET/CT is more appropriate for high-grade poorly differentiated G3 tumors, which generally have higher glucose metabolism. ^18^FDG PET/CT has been reported to be 37%–72% sensitive for the detection of these high-grade tumors. In general, findings of ^18^FDG-positive tumors at PET/CT are indicative of unfavorable prognosis (87, 101–104).

#### 4.2.3. Other applications of nuclear medicine in the diagnosis of neuroendocrine neoplasms

MEN1 syndrome may be associated with tumors developing in several sites, more frequently in the parathyroids, GEP tract and pituitary gland (8). In fact, 90% of patients with MEN1 develop primary hyperparathyroidism before the age of 50. Parathyroid imaging is of paramount importance in the management of parathyroid disease and its aim is to localise all sites of excess hormone secretion before surgery. The spectrum of parathyroid imaging comprises single-photon scintigraphy with Tc-99m-Sestamibi, or dual tracer Tc-99m-pertechnetate and Tc-99m-sestamibi with or without SPECT or SPECT/CT. Combination of cross-sectional imaging and molecular imaging enables optimisation of our diagnostic potential, and grants the ability to have concrete structural and functional information in a single investigation (105).

## 5. The use of endoscopy in the diagnostic cascade

Conventional endoscopy is pivotal in detecting and treating NENs in the upper or lower GI tract (106). More recently, the introduction of EUS in clinical practice enhanced out diagnostic potential. Lastly, small bowel capsule endoscopy (SBCE) and balloon enteroscopy have also emerged as novel and helpful techniques.

### 5.1. Conventional endoscopy in neuroendocrine tumors

Upper gastrointestinal endoscopy with careful appraisal of tumors and background gastric mucosa is still the gold standard in diagnosing gastric and duodenal NENs. In the case of gNENs, gastroscopy establishes the diagnosis. It is critical that multiple biopsies are taken from the antrum and gastric body and fundus, in addition to the largest lesions/polyps (107). dNENs are commonly incidentally found during endoscopy for other indications. As the duodenum is within the reach of conventional endoscopy, histological evaluation and staging, and even curative endoscopic treatment are enabled. Gastroscopy with biopsies can accurately diagnose dNEN, whereas EUS can solidify the diagnosis and complete (local) staging, as discussed later. Some dNENs, such as gastrinomas causing ZES, may be missed on both conventional endoscopy and EUS, and these are diagnosed by hormone assays as described in detail in a previous section (108). Although beyond the scope of this review article, it should be noted that endoscopic management also represents the first line treatment for localized type 1 gNENs, followed by active surveillance. This approach ascertains acceptable oncologic outcomes combined with peri-procedural safety. Classic polypectomy, endoscopic mucosal resection (EMR), or endoscopic submucosal dissection (ESD) modalities are used in clinical practice. Any gNEN > 10mm should be considered for endoscopic treatment, unless suspicion or confirmation exists of muscularis propria invasion or lymph node metastasis, when surgical resection should be considered (106).

In analogy to this, most rNENs are diagnosed incidentally during colonoscopy performed for other indications. rNENs are small, usually 10mm lesions, that resemble benign hyperplastic rectal polyps. Commonly, the endoscopist resects the “hyperplastic polyp”, which proves to be a rNEN on histopathology. This emphatically illustrates why endoscopists should be familiarised with the identification of such lesions. It is also important to be able to distinct NENs from other subepithelial lesions, such as lipomas, which usually do not require treatment. Endoscopic biopsies could be misleading as rNENs are submucosal lesions frequently escaping the diagnosis when biopsies with conventional endoscopy are taken. In addition, random biopsies can cause tissue fibrosis, which may challenge subsequent endoscopic resection (109). This underscores why biopsies should not be taken routinely if a rNEN is strongly suspected, and the critical role of EUS is evident.

### 5.2. Advances in endoscopy in the assessment of the small bowel

#### 5.2.1. The role of small bowel capsule endoscopy in the management of neuroendocrine tumours

The role of SBCE in the diagnosis of SBNENs is not yet well established, in contrast to other endoscopic modalities, and consensus guidelines recommend its use upon local expertise (7). At present, it seems that SBCE may be of value in detecting multifocal SBNENs, in particular in the pre-operative setting to determine the extent of resection. Additionally, the use of SBCE could be considered in cases of metastatic disease of unknown origin before laparotomy. Occasionally, the primary site may remain unclear despite thorough investigations (110). On surgical exploration, most such tumors are detected in the small bowel (111). Nevertheless, one should consider that in NENs of unknown primary, SBCE is 75% sensitive, and only 38% specific compared to laparotomy (112). Additionally, in SBCE contractions of the small bowel or extrinsic compression may give the (false) impression of lesions. Likewise, when a true mass is detected, localization may be inaccurate and additional procedures, such as balloon enteroscopy, may be required for confirmations and pathological evaluation.

#### 5.2.2. The role of balloon enteroscopy in the management of neuroendocrine tumors

The diagnostic yield of bowel enteroscopy (BE) for all small bowel masses varies in different studies (113, 114). In individuals with suspected NENs but inconclusive initial investigations, the diagnostic yield is estimated approximately 33% (115). In a recent study, BE was reported to have 88% sensitivity for the detection of the primary SBNEN, compared to approximately 60% for CT, 54% for MRI, and 56% for somatostatin receptor imaging. In this study, 21.2% of the patients had their primary tumors missed on imaging. Notably, 92.3% of those who had BE, had their primary tumor ultimately identified (116). Similar to SBCE, this modality can identify multifocal NENs pre-operatively. In a retrospective study of subjects who had small bowel resection, pre-operative BE was shown to detect additional lesions in over 50% of patients, compared to 18% with capsule endoscopy (117). The mail limitation of BE would be the fact that it is only or primarily available in referral centres. Considering this, the North American (NANETS) guidelines recommend that multifocal tumors may be most accurately identified at the time of surgery, by examining the entire bowel (118). Overall, the use of BE should be reserved only for centers where it is available and relevant expertise exists.

Overall, BE has the advantage of being an invasive modality, enabling the performance of biopsies, among others. In cases of suspected small bowel lesions, SBCE is usually performed first-line, followed by BE in the case of positive findings (119). Sound relevant evidence or guidance lack in the diagnostic cascade of neuroendocrine neoplasms. Where local expertise does exist, and in an appropriate clinical context as outlined above, these two modalities should have a role, and we believe this is complementary, similar to other small bowel lesions.

### 5.3. The role of endoscopic ultrasound

The advent of EUS revolutionised the field. In particular, it enables detection of previously unidentified tumors; contributes to staging of GEP-NETS, and finally permits tissue diagnosis and histological evaluation.

#### 5.3.1. Endoscopic ultrasound and pancreatic neuroendocrine neoplasms

In particular, it seems that EUS is the most sensitive method for pNENs, being 82%–93% sensitive and 86%–95% specific in this context (120–122). In a series of studies involving more than 200 patients, the detection rate of EUS was ranging from 75 to 97% (55, 99, 100, 123–128). Notably, in a recent review by Ishi et al.*,* tumor grading between EUS-FNA and surgical samples showed a concordance rate of 77.5% (95% CI = 0.59–0.71, *p* < 0.01). (129). Likewise, intraoperative US (IOUS) was also shown to sensitive for pNEN, having a detection rate of 74 to 96% (99, 130–132).

#### 5.3.2. Endoscopic ultrasound and gastroduodenal neuroendocrine neoplasms

For dNENs and lymph node metastases, the detection rate of EUS was 63% in 2 studies comprising 59 patients (99, 126). Forceps biopsy during standard endoscopic examination can diagnose most dNENs, and not uncommonly, endoscopy can offer a curative option for small sporadic dNENs. Nevertheless, for larger tumors over 1 cm, local staging by EUS is recommended before resection. Duodenal NENs are typically submucosal lesions, but they can rarely extend beyond this layer. EUS can accurately establish the degree of submucosal involvement. EUS can accurately assess locoregional lymph node metastases, and this is of particular importance as dNENs can be associated with such metastases in up to 40%–60%, especially gastrinomas. All the above are critical to determine optimal treatment, and candidacy for endoscopic or surgical treatment. Lastly, the pancreas can also be fully interrogated for small tumors, that can be linked to MEN-1 (133–135).

Lastly, gNENs can be classified into three subtypes: type 1 g-NETs which are the most frequent and develop due to hypergastrinaemia in the context of autoimmune atrophic gastritis; type 2 that are linked to increased gastric secretion, in the context of gastrin-secreting tumors, often in patients with MEN-1 and as part of a ZES; lastly, type 3 tumors are sporadic and usually poorly differentiated, mimicking malignant neoplasms of the stomach (136). EUS is recommended for type 1 gNENs > 1 cm prior to endoscopic resection. Similarly, for patients suspected to have type 2 neoplasms, this modality assesses for the presence of dNENs or pNENs. Lastly, for the assessment of type 3 gNENs, EUS evaluates the depth of invasion into the mucosal layers or beyond, and the presence of lymph nodes in the gastro-hepatic and peri-gastric areas (137).

#### 5.3.3. Other application of endoscopic ultrasound

Regarding the role of EUS in the management of rNENs, according to the current ENETS consensus, EUS should follow endoscopic evaluation of a suspected rNEN (138). EUS can accurately assess tumor size, depth of invasion and locoregional lymph node metastases. This can be of paramount importance when determining surgical candidates, and can assist in determining appropriate treatment (109, 139).

## 6. Future perspectives in neuroendocrine neoplasms diagnostics

### 6.1. Novel biomarkers for the diagnosis of neuroendocrine neoplasms

Most of the aforementioned biomarkers, widely used to date, fail to capture the biologic complexity of a NEN; and even the historical Ki67 appears to have some limitations. There is an emerging need to embrace advances in the field and integrate molecular genomic tools into clinical practice. The argument is that contrary to the current biomarkers, multianalyte analysis assess the tumor molecular genomic mechanism. Multianalyte biomarkers include tumor-derived components such as ctDNA, circulating tumor cells (CTCs), miRNA, extracellular vesicles, and “tumor-educated” platelets (140). Key novel biomarkers are summarised in Table 4.

**Table 4:** Novel biomarkers for the diagnosis of neuroendocrine neoplasms (NENs).

Biomarker	Function	Role	Strengths
**NETest**	– mRNA genomic biomarker – Measures a series of transcripts in the blood – Results are expressed as an activity index (NETest score)	– May have a role in the diagnosis, assessment of the effectiveness of surgery, monitoring therapeutic efficacy [including Peptide Receptor Radionuclide Therapy (PRRT)]	– Significant diagnostic accuracy
**Circulating tumor cells (CTCs)**	– Tumor cells shed from the primary tumor or metastasis loci and intravasate into the peripheral blood circulation system	– May have a role as accurate prognostic markers	– Optimal CTC threshold to predict PFS and OS in metastatic pNENs and SB-NENs studied – These thresholds can stratify patients in clinical practice and clinical trials

#### 6.1.1. NETest

The NETest is the most successful multianalyte biomarker assessed to date in the management of NENs. This mRNA genomic biomarker measures a series of relevant transcripts in the blood, which is considered the biological signature of the neoplasm. It is considered a liquid biopsy procedure, assessing the circulating expression level of genes involved in oncogenesis, cell proliferation, signalling and metastasis formation through a peripheral blood real-time polymerase chain reaction (RT-PCR). The respective results are expressed as an activity index (NETest score), ranging between 0 and 100. A score ranging from 21 to 40% represents “stable” disease, whereas a score > 40% reflects “progressive” disease (140). Oberg et al. in their recent meta-analysis reported that the diagnostic accuracy of this tests is approximately 96%. The diagnostic accuracy of this test to differentiate between stable and progressive disease was reported to be between 84.5% and 85.5%. The NETest was 91.5%–97.8% accurate as a marker of natural history and 93.7%–97.4% accurate as an interventional/response biomarker (141).

PRRT is a very effective treatment modality for patients with metastatic and/or inoperable NENs. A radionuclide linked to a SSA is used, and this allows to accurately deliver radiotherapy to somatostatin receptor–expressing neoplasms, such as the great majority of NENs (142). Bodei et al. prospectively evaluated NETest as a surrogate biomarker for Response Evaluation Criteria in Solid Tumors (RECIST). Notably, in over a hundred subjects assessed, NETest significantly decreased in patients who responded to treatment, as per RECIST criteria, and remained elevated in those who did not “respond” to PRRT. Notably, the reported accuracy of treatment response was 98% (143). Similar to the NETest, NEN transcript expression in blood integrated with tumor grade provides a PRRT predictive quotient (PPQ) which also stratifies patients who “respond” to PRRT from those who do not. PPQ response prediction was accurate in 97% with a 99% accurate positive and 93% accurate negative prediction. NETest significantly decreased in PPQ-predicted “responders” and remained elevated or even further increased in PPQ-predicted patients who did not respond to treatment. Interestingly, CgA did not correlate that well with the outcome of PRRT, decreasing in only 38% of treatment “responders” (143).

#### 6.1.2. Circulating tumor cells

CTC measurement has also been assessed in several malignancies. In particular, the CellSearch platform was approved by the US Food and Drug Administration for use in several malignancies, after trials reported the prognostic value of CTCs at defined thresholds (144–146). Although CTCs are also detectable in patients with NENs, studies on the measurement of CTCs in the diagnosis of NENs are not equally enthusiastic (140). On the contrary, CTCs appear more accurate as prognostic markers (147, 148). Interestingly, Mandair et al. defined optimal prognostic CTC thresholds in pNENs and midgut NENs. They used CellSearch to enumerate CTCs in almost 200 subjects with metastatic NENs, as above. These subjects were then followed-up for at least 3 years or until death. CTCs were detected in 33% of patients with pNEN and 51% of midgut NENs. In the multivariate Cox hazard regression analysis for progression free survival (PFS) in subjects with pNEN, 1 or greater CTC had a hazard ratio (HR) of 2.6, whereas 2 or greater CTCs had an HR of 2.25 in midgut NENs. In the multivariate Cox hazard regression analysis for overall survival (OS) in pNEN, 1 or greater CTCs had an HR of 3.16 and in midgut NENs, 2 or greater CTCs had an HR of 1.73 (149).

### 6.2. The impact of radiomics in diagnosis and staging neuroendocrine tumors

The quantitative analysis of medical images data and the extraction of imaging features, also called “radiomics”, represent an emerging approach in personalized medicine and advanced diagnostics, especially for disease characterization or outcome prediction. Similar to other neoplasia, this appears to be a promising tool in the context of NENs. A recent study by Mori et al. evaluated preoperative CT radiomic features as a predictor of tumor grade, the presence of lymph nodes metastases or distant metastases, and microvascular invasion of pNENs. This retrospective study included over 100 patients who underwent surgery for pNEN. They showed that combining few radiomic and clinic-radiological features resulted in presurgical prediction of histological characteristics of pNENs (150). In a different study by Chiti et al., the authors assessed whether CT scan radiomics analysis could predict GEP-NEN grade according to the recent WHO classification; they also concluded that CT-radiomics analysis may contribute to differentiating the histological grade for these tumors (151). Although these studies, among others, illustrate that radiomics may be an invaluable tool in the future, further studies will be required to validate the results.

### 6.3. Recent advents in nuclear medicine in the diagnosis of neuroendocrine tumors

#### 6.3.1. The role of the novel glucagon-like peptide-1 receptors scintigraphy

Insulinomas are rare functional pNENs that secrete insulin arbitrarily, as already discussed. Surgery remains the preferred treatment modality, whenever possible, being linked to cure rate exceeding 98% (152–157). Surgical treatment of insulinomas may be challenged due to difficulties in localizing it using conventional diagnostic modalities, however. In <5%–10% of such individuals, investigations can be negative and non-conclusive (8). Even highly sophisticated and advanced ^68^Gallium-DOTA-SSAs are positive in less than 30% (158). Conversely, insulinomas exhibit a very high density of glucagon-like peptide-1 receptors (GLP-1R). As a result, receptor scintigraphy with radiolabelled GLP-1 receptor analogues is a very promising modality, albeit hampered by its limited availability so far (159–162). In one of the first relevant studies, Christ et al. tested the ^111^In-labeled GLP-1R agonist ^111^In-DOTA-exendin-4 in localizing insulinomas. They found that the GLP-1R scans successfully detected the insulinomas and contributed to the successful surgical resection of insulinomas in all subjects (162).

#### 6.3.2. Dual ^68^Gallium DOTATATE and ^18^F-FDG PET/CT in patients with metastatic gastroenteropancreatic neuroendocrine neoplasms

Recent studies explored whether combining ^68^Gallium-DOTA-SSAs and ^18^F-FDG PET/CT would enhance our ability to determine prognosis. A recent study by Hayes et al. investigated the prognostic utility of a classification system combining the findings of ^68^Ga-DOTATATE and ^18^F-FDG PET/CT and the researchers reported that such a classification tool could indeed correlate with prognosis (163). In addition, Panagiotidis et al. investigated whether ^68^Ga-DOTATATE and ^18^F-FDG PET/CT could influence treatment decisions. The results changed the therapeutic plan in 80.8% of patients. In approximately 21%, ^18^F-FDG PET/CT affected the decision-making, prompting mostly the initiation of chemotherapy. In nearly 50% the treatment cascade was influenced by ^68^Ga-DOTATATE, resulting in consideration of PRRT (164). More recently, a multicentre study assessed and aimed to validate the NETPET score as a prognostic biomarker in metastatic GEP-NENs. The combination of ^68^Gallium-DOTA-SSAs and ^18^F-FDG PET/CT, i.e., “dual PET imaging”, provides a comprehensive overview of the status of the disease. The NETPET score, a 5-point scoring system for dual PET reporting in subjects with metastatic NENs, summarises the information provided by the two modalities into a single parameter. The NETPET score correlated with histological grade (*p* < 0.001), and importantly it was significantly associated with overall survival and time to progression on univariate and multivariate analysis (*p* < 0.01) (165).

### 6.4. Decoding the genetic and molecular profiles of neuroendocrine neoplasms

The advent of pre-clinical models appears to be promising for the design, assessment and evolution of genuinely tailored personalised treatment. In particular, primary culture cells originating from solid neoplasms, have gained significant importance in individualised anti-cancer treatment. In analogy to this, patient-derived xenografts (PDXs) in mice represent an *in vivo* model for the development of individualised precision medicine. To produce PDXs, tumors collected following surgical resection or biopsy, are inoculated as pieces or single-cell suspensions subcutaneously usually into the flank of an animal model (166). More recently, zebrafish PDX (zPDX) emerged as a promising option (167). In the former case, the main advantage is the potential to assess the efficacy of different anti-tumor treatment options in a short time, and also to perform preliminary pre-clinical studies for the identification of novel molecular targets (168, 169). In the latter case, it is anticipated that the effects of antitumor compounds on tumor-induced angiogenesis, invasiveness, metastatic dissemination and tumor cell proliferation can be assessed within very few days after implantation.

Although some of the novel revolutionary techniques presented in the current review are currently in the developmental pipeline, it is surely an insight into the future and illustrate the major paradigm shift currently taking place in medical oncology. Accurate and early diagnosis is critical, whereas there has been some progress, and we definitely believe that the research focus should be in establishing robust prognosticators and prognostic scores.

## 7. Conclusions

It is evident that NENs are complex and heterogeneous tumors, and as such require a sophisticated diagnostic approach. This is critical, as early and accurate diagnosis and staging largely influence prognosis and patient outcomes. Pathology still represents and will likely remain the gold standard in the foreseeable future. Likewise, cross-sectional imaging is still the backbone in the diagnosis and staging of these tumors, and in addition, the advances in CT and MRI have also improved the diagnostic yield. Nevertheless, the genuine revolution in the field follows the advances in nuclear medicine, and the emergence of novel biomarkers assessing the tumor molecular genomic mechanisms. The former, comprising ^68^Ga-DOTA PET/CT exhibits unprecedented diagnostic accuracy, and has been shown to influence and update management in a significant number of patients (97, 164), limited primarily by its high-cost and consequent limited availability. Likewise, multianalyte biomarkers appear promising tools also leading to new horizons. The NETest in particular provides accurate information about the diagnosis, completeness of surgical resection and the presence of residual disease in patients with NENs; it can also predict the therapeutic efficacy of SSAs and PRRT; and lastly it is standardized, reproducible and not influenced by age, gender, ethnicity, fasting or other medications (141, 170, 171). It is evident that we do live in exciting times. Our deeper understanding of these rare neoplasms, the progress already made in the diagnosis and treatment of NENs, and finally these new promising developments, all bring us one step closer to tailored treatment and improved outcomes.
